# Clinical and echocardiographic predictors of left ventricular remodeling following anterior acute myocardial infarction

**DOI:** 10.6061/clinics/2021/e2732

**Published:** 2021-05-27

**Authors:** Caroline Ferreira da Silva Mazeto Pupo da Silveira, Karina Nogueira Dias Secco Malagutte, Bruna Franco Nogueira, Fabrício Moreira Reis, Cássia da Silva Antico Rodrigues, Daniele Andreza Antonelli Rossi, Katashi Okoshi, Rodrigo Bazan, Luis Cuadrado Martin, Marcos Ferreira Minicucci, Silméia Garcia Zanati Bazan

**Affiliations:** I Departamento de Medicina Interna, Faculdade de Medicina, Campus Botucatu, Universidade Estadual Paulista Julio de Mesquita Filho (UNESP), Botucatu, SP, BR.; II Departamento de Neurologia, Faculdade de Medicina, Campus Botucatu, Universidade Estadual Paulista Julio de Mesquita Filho (UNESP), Botucatu, SP, BR.

**Keywords:** Ventricular Remodeling, Acute Myocardial Infarction, Echocardiography, Predictors

## Abstract

**OBJECTIVES:**

Coronary artery disease is the primary cause of death and is responsible for a high number of hospitalizations worldwide. Ventricular remodeling is associated with worse prognosis following ST-segment elevation myocardial infarction (STEMI) and is a risk factor for ventricular dysfunction and heart failure. This study aimed to identify the predictors of ventricular remodeling following STEMI. Additionally, we evaluated the clinical, laboratory, and echocardiographic characteristics of patients with anterior wall STEMI who underwent primary percutaneous intervention in the acute phase and at 6 months after the infarction.

**METHODS:**

This prospective, observational, and longitudinal study included 50 patients with anterior wall STEMI who were admitted to the coronary care unit (CCU) of a tertiary hospital in Brazil between July 2017 and August 2018. During the CCU stay, patients were evaluated daily and underwent echocardiogram within the first three days following STEMI. After six months, the patients underwent clinical evaluation and echocardiogram according to the local protocol.

**RESULTS:**

Differences were noted between those who developed ventricular remodeling and those who did not in the mean±standard deviation levels of creatine phosphokinase MB isoenzyme (CKMB) peak (no remodeling group: 323.7±228.2 U/L; remodeling group: 522.4±201.6 U/L; *p*=0.008) and the median and interquartile range of E/E’ ratio (no remodeling group: 9.20 [8.50-11.25] and remodeling group: 12.60 [10.74-14.40]; *p*=0.004). This difference was also observed in multivariate logistic regression.

**CONCLUSIONS:**

Diastolic dysfunction and CKMB peak in the acute phase of STEMI can be predictors of ventricular remodeling following STEMI.

## INTRODUCTION

Acute myocardial infarction (AMI) is diagnosed when there is evidence of myocardial necrosis along with clinical signs and symptoms consistent with myocardial ischemia (1).

A majority of AMIs occur because of coronary artery atherosclerosis, usually, with overlapping thrombosis. Non-atherosclerotic causes of AMI include vasculitis, trauma, coronary spasm, cocaine, imbalance between oxygen supply and demand, and congenital coronary anomalies (2).

Some of the established criteria for AMI diagnosis are the following: increased cardiac biomarkers (*e.g.*, troponin); ischemic symptoms; ST deviation and T-wave inversion, new left bundle branch block, or pathological Q-wave on electrocardiogram (ECG); loss of viable myocardium or new alterations in segmental contractility on imaging; detection of intracoronary thrombus on angiogram or autopsy; and cardiac arrest with ischemia-like symptoms and ECG alterations (1).

ST-elevation AMI (STEMI) is identified when the previously described AMI criteria are met along with ST elevation or new left bundle branch block (1).

Coronary artery disease is the leading cause of death worldwide (3). In the last few years, the incidence of STEMI has decreased and that of non-STEMI has increased (4). Recent studies have demonstrated a decrease in short-term and long-term mortality following STEMI (5) with a reported mortality rate of 12% at 6 months (greater in high-risk patients) (6). According to 2014 data from DATASUS, AMI was the leading cause of death in Brazil, which was in agreement with the data from the rest of the world (7).

Following STEMI, the heart undergoes a remodeling process that occurs at genetic, molecular, cellular, and interstitial levels (8). The main mechanisms of remodeling are cell death, contractile protein changes, and fibrosis. Clinical repercussions include variations in the organ size, geometry, mass, volume, and function. Remodeling is ultimately a response to damage, even in the non-ischemic myocardium, to adapt to the increased workload. Long-term remodeling is harmful and a risk factor for ventricular dysfunction and heart failure (9).

Among the manifestations of ventricular remodeling following STEMI, left ventricular (LV) aneurism (10) and ventricular arrhythmias stand out. Ventricular arrhythmias (tachycardia and fibrillation) are associated with fibrotic areas, which complicate the transmission of electric impulses. Ohlow et al. reported that the incidence of ventricular arrhythmias was 4.7% in a German facility following STEMI (11). In addition to being arrhythmogenic, fibrotic areas are devoid of contractile properties, thus, resulting in increased wall stress and LV end-diastolic pressure, impaired relaxation, and ultimately, cavity dilatation and loss of contractile capacity (12). Clinically, such processes are considered to be diastolic and systolic dysfunctions.

Ventricular dysfunction can be easily evaluated using an echocardiogram. Diastolic dysfunction is diagnosed based on alterations in the E/A ratio (E wave represents early ventricular filling and A wave represents atrial contraction), E/E’ ratio (E’ represents tissue Doppler early ventricular filling), or an increase in the left atrial diameter, which indicates an increase in LV end-diastolic pressure. In contrast, systolic dysfunction (*i.e.*, alterations in segmental contractility that is commonly seen in STEMI) is better evaluated based on LV ejection fraction (EF) using the Simpson method (13).

In terms of the treatment, the patient must undergo reperfusion therapy as soon as possible following STEMI. The gold-standard procedure is percutaneous coronary intervention (PCI), also called primary angioplasty, with or without coronary stenting (14,15). PCI may be performed safely up to 12 hours after the diagnosis of STEMI; following this duration, benefits of PCI can be observed in those with persistent pain or evidence of myocardial ischemia for up to 24 hours (14,15). After 24 hours, there is no evidence of benefit of PCI in stable patients in terms of new events or LV function in comparison to optimized medical therapy (16). When the duration between the diagnosis and PCI is greater than 90 minutes, fibrinolysis should be performed. If a patient does not meet the reperfusion criteria, rescue angioplasty is performed. The sooner the intervention is performed, the greater is muscle preservation and the lesser is the infarction area and residual ventricular dysfunction.

In addition to PCI, drugs are also important in the treatment of STEMI. Some studies suggest early administration of drugs prior to any intervention (17), and some drug classes are associated with reversal of remodeling, such as angiotensin-converting enzyme inhibitors (ACEIs), angiotensin II receptor blockers (ARBs), aldosterone inhibitors, and beta blockers, which act on the myocardium and contribute to the recovery of contractility and cardiac function (18).

Chen et al. reported that patients with STEMI presented with diastolic dysfunction in early evaluations irrespective of the duration between STEMI and PCI (18). Shacham et al. noted an association between the E/E’ ratio and an increase in diastolic and systolic volumes after 4-8 months (19).

It is known that ventricular remodeling is associated with worse prognosis following myocardial infarction. Therefore, the hypothesis of the present study was that additional prognostic information may help identify patients at a greater risk of ventricular remodeling. The aim of this study was to identify predictors of ventricular remodeling following AMI.

## PATIENTS AND METHODS

### Study design

This prospective, observational, and longitudinal study was performed in the coronary care unit (CCU) of the Clinics Hospital of Botucatu Medical School (HC-FMB-UNESP) and included patients with left anterior wall STEMI.

All procedures were approved by the Research Ethics Committee (CEP) of Botucatu Medical School (n° 2.102.000), and all patients were informed about the procedures and signed a consent form before they were included in the study.

### Patients

**Figure 1:**
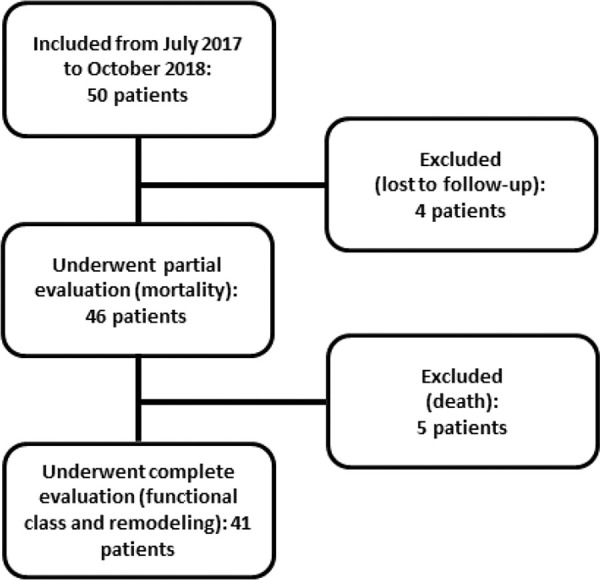
Flowchart of patient inclusion.

All eligible patients in the CCU of the Clinics Hospital of the Botucatu Medical School between July 2017 and October 2018 were included; overall, 50 patients were included. Five of these patients died, and four were lost to follow-up at 6 months. Figure 1 shows the flowchart of study inclusion.

### Inclusion criteria

Enrolled patients included consecutive patients of both sexes who were over 18 years of age, were diagnosed with LV anterior wall STEMI, and underwent primary angioplasty between July 2017 and October 2018.

Anterior wall STEMI was diagnosed based on history of chest pain of 20 minutes to 12 hours and ST elevation ≥2 mm in at least two precordial leads or the presence of an assumingly new left bundle branch block. AMI diagnosis was confirmed by an increase in cardiac enzyme levels to at least twice the upper limit (1).

### Exclusion criteria

Patients who were excluded from the study were those with age <18 years, congenital heart disease, significant valvulopathies, hypertrophic or infiltrative cardiomyopathies, previous myocardial infarction, atrial fibrillation, pacemaker, pregnancy, inappropriate echocardiogram window, liver failure, cancer, and chronic use of immunosuppressors or anti-inflammatory drugs. Additionally, patients who were lost to follow-up were excluded. Patients who died during the 6-month follow-up period were included in the analysis.

## METHODS

According to the CCU regimen, patients were evaluated daily and underwent the first echocardiogram 2-3 days after AMI. The follow-up period was 6 months after hospital discharge; the patients underwent clinical evaluation and echocardiogram at 6 months according to the local protocol.

### Clinical evaluation

Clinical evaluations were performed at two time points; one during hospitalization and the other at 6 months after anterior wall STEMI based on anamnesis and physical examination.

The clinical variables assessed during admission were the following: age, sex, symptoms, chest pain duration (until emergency room admission), coexisting comorbidities, cardiovascular risk factors (hypertension, diabetes, dyslipidemia, smoking, and family history of coronary artery disease), continuous use of medications prior to admission, heart rate, systemic blood pressure, and signs of systemic and pulmonary congestion.

Possible in-hospital clinical complications following AMI included post-infarction angina, heart failure, pericarditis, arrhythmias, cardiogenic shock, and death.

Clinical examination at 6 months included evaluation of dyspnea and chest pain, continuous use of drugs after discharge, heart rate, systemic arterial pressure, and signs of congestion.

### Laboratory evaluation

Blood samples were collected according to the routine protocol of the CCU. Upon admission, electrolytes, urea, creatinine, and blood counts were assessed. Total creatine phosphokinase (CPK) and MB isoenzyme (CKMB) were evaluated upon arrival and every 6h until early decline in their levels. Troponin I level was evaluated upon arrival and at 90 min later. Blood levels of glucose and glycosylated hemoglobin, low-density lipoprotein (LDL), high-density lipoprotein (HDL), total cholesterol, and triglycerides were also assessed during the in-hospital stay.

### Primary coronary angioplasty evaluation

The following variables were documented for analysis: duration between the onset of chest pain and reperfusion; the extent of coronary artery disease (number of arteries with at least 70% obstruction); infarction-related artery (IRA) flow after the intervention as determined by *Thrombolysis in Myocardial Infarction* (TIMI) classification (0: complete IRA obstruction; 1: the contrast enters until it reaches the obstruction point without completely opacifying the artery; 2: opacification of the whole artery but with slow flow; 3: complete perfusion of IRA with normal flow (20)); myocardial reperfusion after primary angioplasty using myocardial blush grade (0: contrast failed to enter microcirculation; 1: slow entry of contrast in the microcirculation without proper elimination; 2: delayed entry and exit of contrast from the microcirculation; 3: normal entry and exit of contrast from the microcirculation (21)).

### Echocardiographic evaluation

Echocardiograms were performed during hospitalization (2-3 days after admission) and at 6 months using GE Vivid S6^®^ (General Eletric Medical Systems, Tirat Carmel, Israel) with a multifrequency ultrasonic transducer of 2.0-3.5 MHz and an image recording system. Images were obtained and analyzed according to the recommendations of the American Society of Echocardiography (22).

Morphometric variables included the following:

- Left atrial maximum diameter (mm): LA;
- Left atrial volume (LAV, mL), obtained using the Simpson method;
- Left atrial volume index (LAVI, mL/m^2^): LAV normalized according to body surface area (BSA);
- LV diastolic and systolic diameters (mm): LVDD and LVSD, respectively;
- Interventricular septum and LV posterior wall diastolic thickness (mm): IVS and PW, respectively;
- Relative wall thickness (RWT)=(2 x PW)/LVDD;
- LV mass (LVM, g)=0.8×{1.04×[(IVS+PW+LVDD)^3^- LVDD^3^]}+0.6; and
- LVM index (LVMI, g/m^2^)=MVE/BSA.

Systolic function variables included the following:

- LV EF, obtained using the Simpson method and
- Fractional shortening (%ΔD)=[(LVDD-LVSD)/LVDD]x100.

**Figure 2:**
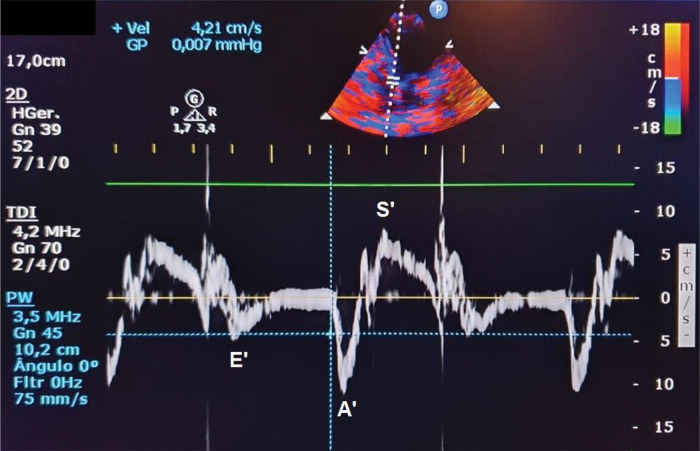
Septal tissue Doppler in the apical window four-chamber view to assess the velocity of mitral annular systolic (S’) and diastolic excursion in the early filling phase (E’) and atrial contraction phase (A’).

Diastolic function variables included the following:

- Ventricular early filling phase peak velocity (E wave, cm/s): obtained using spectral Doppler of transmitral diastolic flow;
- Atrial contraction phase peak velocity (A wave, cm/s): obtained using spectral Doppler of transmitral diastolic flow;
- E/A ratio;
- LV isovolumetric relaxation time (IVRT, ms), corresponding to the period between the end of ventricular ejection and the beginning of diastolic transmitral diastolic flow;
- E wave deceleration time (EDT, ms), corresponding to the time between the initial transmitral flow peak velocity and its extrapolation to baseline;
- Velocity of mitral annular diastolic excursion on tissue Doppler in the early filling phase was obtained from the mean of septal and lateral sites (E’) (Figure 2);
- E/E’ ratio.

LV remodeling was defined as an increase in LV systolic and/or diastolic volume of at least 15% within 6 months of follow-up (23). The volume was obtained using the Teichholz formula [(7xD^3^)/(2.4+D)] based on LV systolic and diastolic diameters (D).

### Statistical analysis

Continuous variables with normal and non-normal distributions are presented as mean and standard deviation or median and 25^th^ and 75^th^ percentiles and were compared using the Student’s *t*-test and Mann-Whitney test, respectively. Categorical variables were analyzed using the chi-squared test or Fisher’s exact test.

To evaluate the association of variables with remodeling outcomes within 6 months, we used multivariate logistic regression (24). The variables with statistical significance in univariate analysis were included with significance level set at 5%.

For continuous variables that presented differences in multivariate logistic regression, receiver-operating characteristics (ROC) curves were designed to define the thresholds.

## RESULTS

We evaluated 50 patients with anterior wall STEMI who underwent primary angioplasty. The overall mean age was 61.2±9.8 years, and 84% were male. Four patients were lost to follow-up, and five (10%) died within the 6-month follow-up period. Therefore, 41 patients were re-evaluated clinically and underwent a second echocardiography at 6 months after AMI. Of these, 36.6% presented with ventricular remodeling.

Table 1 summarizes the clinical, demographic, and laboratory characteristics of the patients who were divided into the following two groups according to ventricular remodeling: yes (n=15) and no (n=26). There was homogeneity between the groups in terms of the age, sex, comorbidities, risk scores, laboratory test results at admission, angiographic variables, and in-hospital and post-discharge complications. The in-hospital complications were the following: cardiogenic shock, non-sustained ventricular tachycardia, acute stent thrombosis, LV aneurysm with thrombus, altered renal function, pneumonia, and acute pulmonary edema. Post-discharge complications were the following: septic shock, acute pulmonary edema, death, high-risk unstable angina, and high digestive hemorrhage. There was a significant difference between the groups in the peak value of CKMB (No group: 323.7±228.2 U/L; Yes group: 522.4±201.6 U/L; *p*=0.008).

Table 2 summarizes the initial echocardiographic data. The data were similar between groups except the final systolic and diastolic diameters and volumes, which were used for defining ventricular remodeling, and the E/E’ ratio, which was greater in the group with ventricular remodeling (No group: 9.20 [8.50-11.25]; Yes group: 12.60 [10.74-14.40]); *p*=0.004).

Table 3 summarizes the medications administered at hospital discharge and the criteria of clinical re-evaluation after 6 months with no difference between the groups.

**Figure 3:**
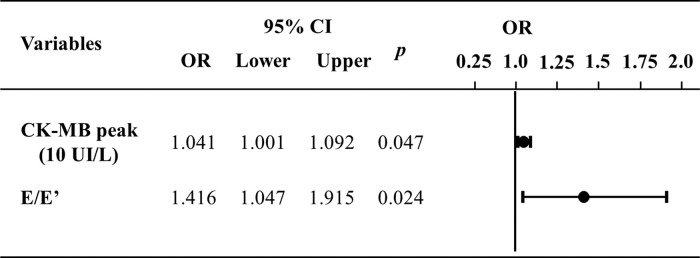
Multivariate logistic regression to predict ventricular remodeling at 6 months. E: transmitral flow peak velocity in early filling phase; E’: the speed of mitral annular diastolic excursion on tissue Doppler in the early filling phase; CKMB, creatinine kinase MB isoenzyme; CI, confidence interval; OR, odds ratio.

Figure 3 illustrates the multivariate logistic regression analysis of variables that demonstrated differences between the groups. It shows that the difference between the groups remained for the prediction of ventricular remodeling at 6 months.

**Figure 4:**
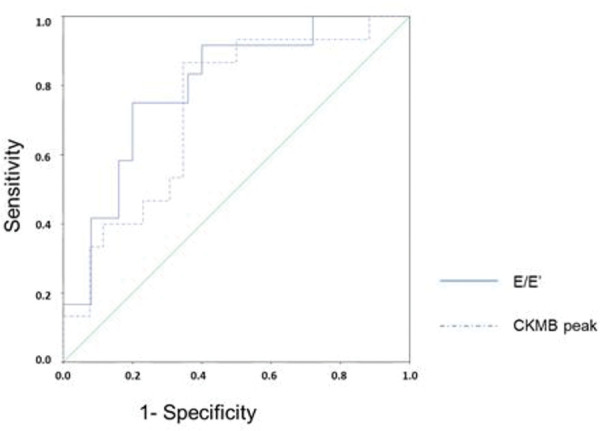
ROC curve for predicting ventricular remodeling based on the E/E’ ratio and CKMB peak. ROC, receiver-operating characteristics; E: transmitral flow peak velocity in early filling phase; E’: the speed of mitral annular diastolic excursion on tissue Doppler in the early filling phase; CKMB, creatinine kinase MB isoenzyme.

Figure 4 presents the ROC curves for the diagnosis of ventricular remodeling in relation to the CKMB peak level and E/E’ ratio. The area under the curve for the CKMB peak level was 0.733 (95% confidence interval, CI: 0.575-0.892), and that for E/E’ ratio was 0.797 (95% CI: 0.646-0.947). The CKMB peak level of 378 U/L had sensitivity of 86.7% and specificity of 65.4% in predicting ventricular remodeling. The threshold for the E/E’ ratio was 11.56 with a sensitivity of 75% and specificity of 80% for early detection of ventricular remodeling. After combining these two variables, the sensitivity fell to 66.7% whereas the specificity increased to 84.0% in predicting ventricular remodeling. When either of the variables was used, either CKMB peak ≥378 U/L or E/E’ ratio ≥11.56, the sensitivity was 100% but specificity decreased to 60.0%.

## DISCUSSION

The aim of our study was to compare the parameters of patients with STEMI in the acute phase and after 6 months of follow-up to identify predictors of ventricular remodeling.

Data from the acute phase provided valuable information about these patients. For instance, Chen et al. found diastolic dysfunction even after early reperfusion (12). Barberato et al. also defined the E/E’ ratio as a predictor of LV remodeling following AMI (25). Shacham et al. observed that E/E’ ratio >15 in the acute phase was related to ventricular remodeling (19). Other studies noted that the E/E’ ratio was associated with mortality following STEMI (26,27). In the present study, E/E’ ratio of 11.56 presented good sensitivity and specificity in predicting ventricular remodeling within 6 months; the sensitivity could be increased in combination with CKMB peak ≥378 U/L. Van Melle et al. obtained the average tissue Doppler value of six myocardial sites (from three apical windows) and did not find a relationship with ventricular remodeling but with infarction size and the worsening of EF (28). In contrast, reduction in E/E’ ratio was associated with smaller dimensions of the left ventricle. Such alterations, though not completely understood, could be explained by the stunned myocardium, which can be present even within a few hours of pain and can lead to rigidity of the ventricular wall. This finding was also observed in the present study; a higher E/E’ ratio was noted in the group with remodeling compared to that in the group without remodeling (Table 2).

Hellawell et al. findings on reverse remodeling reinforce the idea that the benefit of early reperfusion lies in fighting an unfavorable metabolism, showing positive effects in both primary angioplasty and thrombolysis (18). There are reports of improvement in ventricular diameter after 6 months of follow-up (29). The benefit was even greater in patients treated with primary angioplasty, which was performed up to 12 hours (mean, 5.5±7 hours) after the onset of pain, with an increase of 10% in EF in 39% of patients. The study also demonstrated that the maintenance of reverse remodeling is associated with encouraging long-term results (30). In contrast, late reperfusion, reperfusion performed over 12 hours after the onset of pain, does not bring such evident benefits and results in little increase in EF and only minor reduction in ventricular volumes (31). The present study did not employ late reperfusion once our hospital’s protocol was revised to recommend that primary angioplasty should be performed up to that threshold, although in selected cases, such as hemodynamic instability or pain persistence, late perfusion may be executed. Therefore, patients were divided according to reperfusion time of less than and greater than 6h after the onset of pain. The reperfusion time did not influence ventricular remodeling at 6 months in this study. These data support the concept that the 12-hour period provides good results in the medium term, and the conditions of the patients who underwent primary angioplasty 12 hours after the onset of pain evolved without ventricular remodeling. Considering the data reported in the literature, we believe that greater differences would have been observed if we had enrolled patients who underwent late reperfusion or had included a larger sample size.

The CKMB peak level was closely related to the infarction size and was related to ventricular remodeling (Table 1) as previously reported (32).

In our study, there was no relationship between the remaining variables and ventricular remodeling. We found in the literature that neutrophils might be altered once the inflammatory reaction following AMI increases the tissue damage. In contrast, there are experimental findings that point to the importance of neutrophils in the repair processes following infarction since their absence in knockout rats is related to worsening in ventricular function, increase in fibrosis, and heart failure (33).

Our study highlights the importance of delay in forwarding patients to our hospital, which can surpass the recommended 12 hours for primary angioplasty. This setback may be due to the patients themselves who are often deprived of health education and neglect the initial AMI symptoms or the regional emergency health assistance, which often surpassed the 10 minutes recommended for STEMI diagnosis. There were also cases of patients who were referred in a timely manner but had already received thrombolytic therapy and were candidates for rescue angioplasty, which was considered an exclusion criterion. These factors limited the sample size, which was initially estimated to be 65 people per year. The economic status of the national health system should also be noted because it limited some biochemical examinations during some periods, notably that of troponin, which is essential in the characterization of these patients.

Some previously established concepts were reinforced, such as the correlation of the infarction size, defined in this study by CKMB peak, and diastolic dysfunction in the acute phase, defined as an increase in the E/E’ ratio, with ventricular remodeling as well as the efficacy of primary angioplasty within 12 hours to avoid cardiac dilatation. It was then possible to contextualize the literature findings in the real-life situation of a university hospital. Once we have established values for E/E’ ratio and CKMB peak with good sensitivity and specificity in predicting ventricular remodeling, we can identify patients who should be re-evaluated earlier.

It is noteworthy that the people within the region still require health education so that patients can seek help earlier and hospitals can begin medications and forward them to our center as soon as possible for the gold-standard treatment.

## CONCLUSION

The results of the present study allowed us to identify diastolic dysfunction and CKMB peak in the acute phase of STEMI as predictors of subsequent ventricular.

## AUTHOR CONTRIBUTIONS

Silveira CFSMP contributed to the literature search, study design, data collection, analysis and interpretation, and manuscript drafting. Malagutte KNDS, Nogueira BF and Reis FM contributed to the literature search, data collection, data analysis and interpretation, and manuscript drafting. Rodrigues CSA, Rossi DAA, Okoshi K, Bazan R and Martin LC contributed to the literature search, data analysis and interpretation, and manuscript drafting. Minicucci MF, and Bazan SGZ contributed to the literature search, study design, data analysis and interpretation, and manuscript drafting.

## Figures and Tables

**Table 1:** Clinical, demographic, and laboratory characteristics of patients with STEMI and ventricular remodeling.

Variable	Ventricular remodeling		*p*-value
	No (n=26)	Yes (n=15)	
Age (years)	60.6±9.9	59.7±8.7	0.768
Male	84.6 (22)	80.0 (12)	0.693
Aborted SD	3.8 (1)	20.0 (3)	0.130
Diabetes	34.6 (9)	26.7 (4)	0.734
Hypertension	57.7 (15)	60.0 (9)	0.854
Dyslipidemia	26.9 (7)	40.0 (6)	0.492
Smoking	61.5 (16)	66.7 (10)	0.993
Previous CAD	0 (0)	33.3 (5)	0.139
Family history	11.5 (3)	13.3 (2)	0.104
Days in CCU >5	34.6 (9)	40.0 (6)	0.937
High GRACE	46.2 (12)	60.0 (9)	0.596
TIMI >4	92.3 (24)	100.0 (15)	0.524
Thyropathy	3.8 (1)	13.3 (2)	0.542
Coagulopathy	0 (0)	0 (0)	1.000
Anemia	3.8 (1)	6.7 (1)	1.000
Polyglobulia	15.4 (4)	6.7 (1)	0.636
Creatinine >1.5 mg/dL	3.8 (1)	13.3 (2)	0.543
Neutrophils >10,000	42.3 (11)	33.3 (5)	0.869
Peak CKMB (U/L)	323.7±228.2	522.4±201.6	0.008
Peak CPK (U/L)	3162 (1159-6257)	4817 (3181-7851)	0.163
High blood glucose	69.2 (18)	66.7 (10)	1.000
Elevated liver enzymes	73.1 (19)	66.7 (10)	0.436
Hyperuricemia	15.4 (4)	13.3 (2)	1.000
LDL >100 mg/dL	53.8 (14)	60.0 (9)	0.887
HDL >40 mg/Dl	46.2 (12)	60.0 (9)	0.780
Total cholesterol >190 mg/dL	34.6 (9)	40.0 (6)	0.986
TG >175 mg/dL	19.2 (5)	33.3 (5)	0.449
Angio <6 hours	73.1 (19)	46.7 (7)	0.176
TIMI flow 3	96.2 (25)	100.0 (15)	1.000
Blush 3	61.5 (16)	40.0 (6)	0.507
Thrombus	42.3 (11)	46.7 (7)	0.956
Multiarterial	57.7 (15)	46.7 (7)	0.721
Agrastat	46.2 (12)	33.3 (5)	0.636
Residual lesion	88.5 (23)	60.0 (9)	0.053
In-hospital complications	42.3 (11)	53.3 (8)	0.721
Follow-up complications	11.5 (3)	13.3 (2)	1.000

Categorical variables are expressed as percentages followed by absolute values, and continuous variables are expressed as mean±standard deviation when normally distributed and median and interquartile range (25%-75%) when non-normally distributed. SD: sudden death; CAD: coronary artery disease; CCU: coronary care unit; GRACE: score based on *Global Registry of Acute Coronary Events*; TIMI: *Thrombolysis in Myocardial Infarction;* CKMB: creatine kinase MB; CPK: total creatine phosphokinase; LDL: low-density lipoprotein; HDL: high-density lipoprotein; TG: triglycerides.

**Table 2:** Initial echocardiographic characteristics of patients and ventricular remodeling.

Variables	Ventricular remodeling		*p*-value
	No (n=26)	Yes (n=15)	
LA (mm)	40.5±3.5	40.3±4.9	0.923
LAV (mL)	58.0 (48.9-68.5)	52.0 (45.0-60.5)	0.331
LAVI (mL/BSA)	29.8 (25.5-35.3)	29.7 (24.2-34.7)	0.882
LVDD (mm)	51.5±4.9	49.9±3.9	0.289
LVSD (mm)	36.8±6.0	35.7±5.4	0.561
Diastolic vol (mL)	128.0±28.3	118.5±20.6	0.262
Systolic vol (mL)	59.6±22.5	55.0±18.8	0.510
IVS (mm)	11 (9-13)	10 (9-13)	0.785
PW (mm)	10.9±1.7	10.7±1.7	0.623
Relative wall thickness	0.42 (0.36-0.47)	0.44 (0.38-0.47)	0.597
LVM (g)	211.7±59.5	205.7±61.0	0.419
LVMI (g/m^2^)	117.4±30.4	110.0±31.5	0.483
EF (%)	45.1 (41.9-49.3)	44.0 (39.1-48.6)	0.357
%ΔD	0.29±0.06	0.29±0.07	0.925
TAPSE (mm)	20.9±2.1	21.1±1.9	0.774
RV (mm)	34.4±3.1	32.9±3.4	0.195
E wave (cm/s)	71.4±18.0	61.7±16.4	0.096
A wave (cm/s)	84.6±17.4	80.5±20.9	0.515
E/A	0.74 (0.65-1.15)	0.70 (0.60-0.80)	0.352
IVRT (ms)	117.0 (112.0-123.5)	121.0 (112.5-147.5)	0.501
EDT (ms)	239.5±62.4	231.5±46.8	0.747
E/E’	9.20 (8.50-11.25)	12.60 (10.74-14.40)	0.004

Continuous variables are expressed as mean±standard deviation when normally distributed and as median and interquartile range (25%-75%) when non-normally distributed. LA: left atrial diameter; LAV: left atrial volume; LAVI: left atrial volume index; BSA: body surface area; LVDD: left ventricular diastolic diameter; LVSD: left ventricular systolic diameter; Vol: volume; IVS: interventricular septum diastolic thickness; PW: left ventricular posterior wall diastolic thickness; LVM: left ventricular mass; LVMI: left ventricular mass index; EF: left ventricular ejection fraction by Simpson’s method; %ΔD: left ventricular shortening fraction; TAPSE: tricuspid annular plane systolic excursion; RV: right ventricle; E and A: transmitral flow peak velocity in early filling and atrial contraction phase, respectively; E/A: the ratio between E and A; IVRT: isovolumic relaxation time; EDT: E wave deceleration time; E’: the speed of mitral annular diastolic excursion on tissue Doppler in the early filling phase; E/E’: the ratio between the E and E’ waves.

**Table 3:** Medications at hospital discharge and clinical criteria of re-evaluation according to ventricular remodeling.

Variables	Ventricular remodeling		*p*-value
	No (n=26)	Yes (n=15)	
ACEI	61.5 (16)	60.0 (9)	0.814
ARB	7.7 (2)	6.7 (1)	1.000
Aldosterone inhibitor	30.8 (8)	26.7 (4)	1.000
Beta blocker	96.2 (25)	80.0 (12)	0.130
Nitrate	3.8 (1)	0 (0)	1.000
Trimetazidine	3.8 (1)	0 (0)	1.000
AAS	100.0 (26)	100.0 (15)	1.000
DAPT	100.0 (26)	100.0 (15)	1.000
Diuretic	23.1 (6)	40.0 (6)	0.311
Anticoagulant	15.4 (4)	20.0 (3)	0.693
Insulin	0 (0)	0 (0)	1.000
Digitalis	7.7 (2)	13.3 (2)	0.615
Statin	100.0 (26)	100.0 (15)	1.000
Controlled HR	57.7 (15)	100.0 (15)	0.239
Controlled SBP	96.2 (25)	100.0 (15)	1.000
Controlled DBP	76.9 (20)	93.3 (14)	0.232
Worsening of FC	19.2 (5)	20.0 (3)	1.000

Categorical variables are expressed in percentages followed by absolute values. ACEI: angiotensin-converting enzyme inhibitor; ARB: angiotensin II receptor blocker; AAS: acetylsalicylic acid; DAPT: dual antiplatelet therapy; HR: heart rate; SBP: systolic blood pressure; DBP: diastolic blood pressure; FC: functional class.
